# Exposure to Non-Native Tropical Milkweed Promotes Reproductive Development in Migratory Monarch Butterflies

**DOI:** 10.3390/insects10080253

**Published:** 2019-08-16

**Authors:** Ania A. Majewska, Sonia Altizer

**Affiliations:** 1 Odum School of Ecology, University of Georgia, Athens, GA 30602, USA; 2 Center for the Ecology of Infectious Disease, University of Georgia, Athens, GA 30602, USA; 3 Department of Biology, Emory University, Atlanta, GA 30322, USA

**Keywords:** *Asclepias curassavica*, *Danaus plexippus*, garden, reproductive diapause, physiology, migration, sedentary

## Abstract

Background: North American monarchs (*Danaus plexippus*) are well-known for their long-distance migrations; however, some monarchs within the migratory range have adopted a resident lifestyle and breed year-round at sites where tropical milkweed (*Asclepias curassavica*) is planted in the southern coastal United States. An important question is whether exposure to exotic milkweed alters monarch migratory physiology, particularly the ability to enter and remain in the hormonally-induced state of reproductive diapause, whereby adults delay reproductive maturity. Cued by cooler temperatures and shorter photoperiods, diapause is a component of the monarch’s migratory syndrome that includes directional flight behavior, lipid accumulation, and the exceptional longevity of the migratory generation. Methods: Here, we experimentally test how exposure to tropical milkweed during the larval and adult stages influences monarch reproductive status during fall migration. Caterpillars reared under fall-like conditions were fed tropical versus native milkweed diets, and wild adult migrants were placed in outdoor flight cages with tropical milkweed, native milkweed, or no milkweed. Results: We found that monarchs exposed to tropical milkweed as larvae were more likely to be reproductively active (exhibit mating behavior in males and develop mature eggs in females) compared to monarchs exposed to native milkweed. Among wild-caught fall migrants, females exposed to tropical milkweed showed greater egg development than females exposed to native or no milkweed, although a similar response was not observed for males. Conclusions: Our study provides evidence that exposure to tropical milkweed can increase monarch reproductive activity, which could promote continued residency at year-round breeding sites and decrease monarch migratory propensity.

## 1. Introduction

Insect migrations are diverse, pervasive, and increasingly disrupted by human activities [1,2,3,4]. Physical obstacles and roads can reduce movement, alter migration routes, and directly cause mortality [5,6]. Climate warming is affecting resource phenology and the timing of insect departure and arrival [7,8,9]. Some migratory insects are responding to anthropogenic activities by traveling shorter distances, while others are forming subpopulations that no longer migrate [10]. Declining migratory insect populations and shifts in migratory behavior are especially concerning given the importance of insect migrants in the transport of nutrients and biomass over vast distances and across ecosystems [3], as well as the benefits of migration in allowing some insects to escape from natural enemies [11,12].

Physiological changes associated with migratory behavior in insects commonly occur in response to changes in environmental cues [13,14]. Shorter photoperiods, cooler temperatures, reduced food quality, and changes in moisture, pH, and secondary plant compounds are known to induce insects to prepare for and initiate migration or diapause-related dormancy [15,16]. Milkweed bugs (*Oncopeltus fasciatus*), for example, exhibit a facultative reproductive diapause associated with migratory behavior, triggered by the exposure of immature stages to short photoperiods and cooler temperatures [17,18]. Disruptions to the timing, frequency, or intensity of the cues has the potential to impact insect life history and migration. For instance, higher temperatures led to increased residency of migratory *Lepidoptera* in Europe [19], with some species capable of surviving mild winters in England [20,21]. Because the mechanisms that promote migration in insects can be complex, identifying which human activities are threatening their migrations requires detailed study.

One of the best-studied insect migrations is that of the North American monarch (*Danaus plexippus*), a species that is increasingly impacted by anthropogenic activities. In eastern North America, monarchs emerge at the end of the summer and early autumn in a state of reproductive diapause [1,22,23], and migrate south to wintering sites in central Mexico. Delayed reproduction in monarchs is thought to facilitate the energy storage and greater longevity needed to complete this successful two-way migration [1]. After spending up to five months at the wintering sites, the same monarchs that flew south then mate, and fly north in the spring to recolonize the southern part of their breeding range [24,25]. Resident (non-migratory) populations have been reported since at least the early 2000s along parts of the southern coastal United States [26] in response to the human planting of exotic tropical milkweed (*Asclepias curassavica*). Unlike the estimated 100 milkweed species native to North America [27] that senesce during late summer and fall, tropical milkweed remains in leaf and flowers year-round in mild climates [10,26,28,29]. For this reason, tropical milkweed provides monarch adults with nectar and foliage for oviposition and caterpillar feeding during the times of year when native host plants are largely absent [28]. Resident monarchs show mating and egg laying activity on tropical milkweed throughout the autumn and winter months [26,28], during a time of year when migratory monarchs in Mexico are non-reproductive. This year-round breeding activity is associated with high local densities and greater transmission of the debilitating protozoan parasite *Ophryocystis elektroscirrha* [10,30].

Factors that facilitate the formation persistence of resident behavior in the southern coastal United States require further study. To date, nearly all resident monarch populations in the United States are found at sites with non-native *A*. *curassavica* [10,26,30]. Also known as bloodflower or scarlet milkweed, this species native to central and South America was introduced into the United States, as well other countries across the globe, by the horticulture business as an easy-to-grow ornamental plant that attracts butterflies and other beneficial insects [10,27,31,32,33]. Tropical milkweed is not naturally present in the United States, but has been planted throughout gardens and parks (Figure S1) [27,34], and is available for purchase via popular garden centers (e.g., Lowes) and online stores (e.g., Amazon). In addition to remaining in leaf and flower year-round in areas protected from winter frosts, tropical milkweed is highly attractive to monarchs, yielding higher egg and caterpillar densities compared to native milkweeds [35,36]. This is likely due to tropical milkweed’s high concentration of cardenolide secondary compounds, which monarchs sequester to aid in anti-predator defense through early adulthood [37,38]. Moreover, tropical milkweed is attractive to ovipositing females infected by *O. elektroscirrha* [39], and the concentrated cardenolide compounds are known to lower parasite spore loads for monarchs relative to those that feed on less toxic milkweed species as larvae [40].

Two underlying physiological mechanisms could explain the formation of resident year-round breeding populations in the presence of tropical milkweed in the southern United States. First, tropical milkweed might limit diapause induction during larval development, increasing the probability of the emergence of breeding adults. Previous work on reproductive diapause in monarchs shows that the induction of diapause depends on multiple environmental cues experienced during pre-adult development, including milkweed quality and plant age [13,41]. Specifically, milkweed that is aged and of poor quality tends to result in a higher proportion of monarchs that emerge in diapause [13,41]. Yet, whether diapause induction in monarchs differs between native versus non-native milkweed diets remains unclear. It is possible that the high concentration and diversity of cardenolide compounds in tropical milkweed [42] could affect the hormonal mediation of reproductive diapause in monarch caterpillars. Further, as proposed by Batalden and Oberhauser [28], tropical milkweed might initiate the development of the reproductive system in migratory monarchs (i.e., adults in reproductive diapause). Tropical milkweed encountered by fall migrant monarchs is likely to be an attractive reproductive resource [28]. Past studies suggest that migratory monarchs captured at sites in Texas with tropical milkweed showed reproductive activity and remained at these sites for weeks [28,30], in contrast to migrants captured at sites without tropical milkweed. It is also possible that exposure to any milkweed species as a reproductive resource (i.e., milkweed with good quality foliage), rather than tropical milkweed per se, could cause adult monarchs in diapause to become reproductively active. Since native milkweeds during fall migration tend to be nearing dormancy and poor quality, testing the effect of milkweed species identity versus milkweed presence on monarch reproductive diapause requires controlled experiments.

Here we examine how tropical milkweed exposure during larval and adult stages influences the induction and maintenance of reproductive diapause. If tropical milkweed as a larval diet limits the induction of reproductive diapause, then monarch larvae fed tropical (versus native) milkweed will be more likely to emerge as adults in a reproductively active state. Second, if tropical milkweed exposure increases the chances of reproductive development in fall migrating monarchs (presumably already in a state of diapause), then a higher proportion of migratory monarchs exposed to tropical milkweed will become reproductively active, relative to those exposed to native milkweed or no milkweed. We evaluate these hypotheses via laboratory and field experiments, whereby monarch larvae were fed different milkweed diets in controlled fall-like conditions previously shown to induce reproductive diapause; and wild-caught migratory monarchs were exposed to tropical milkweed, native milkweed, and no milkweed in field cages during the autumn months. We compared reproductive activity by observing mating events and via dissections to evaluate oocyte development in females, and reproductive tract mass in males.

## 2. Materials and Methods

### 2.1. Monarch Migration and Reproductive Diapause

Each fall, monarch butterflies in eastern North America travel as far as 3000 km to high-altitude forests in central Mexico to overwinter [43]. Successful fall migration and survival through the overwintering period are thought to depend in part on reproductive diapause, a physiological state marked by little to no development of reproductive tissues, and lack of mating behavior in monarchs until the following spring [1,23,24,44]. All monarchs sampled early in the wintering period at the Mexico sites have immature reproductive tracts [45], suggesting that long-distance migration and overwintering is more successful with delayed reproduction. However, it is important to note that a proportion of monarchs (10–35%) captured in autumn months along the migratory path show reproductive activity [30,46], and whether these monarchs successfully reach the wintering sites in Mexico is unclear.

As a trans-generational physiological shift, monarchs that emerge in reproductive diapause differ from short-lived adults that breed in the summer [47]. Diapause monarchs show extended adult longevity of up to 8 months, increased allocation to fat reserves, larger wing sizes, higher body mass, and more efficient flight [22,24,45,47,48,49,50], all of which is thought to aid in the completion of their migratory cycle. Previous work indicates that in late summer and early fall, when monarch larvae experience a decreasing photoperiod and cooling average temperatures, the juvenile hormone is present at a low level, causing reproductive diapause [23,41]. Low-quality and older host plants also increase monarch propensity for diapause relative to the case where caterpillars are fed young, high-quality plants [13,41]. Reproductive diapause is commonly assessed via behavioral observations of mating activity, as well as by dissections of adults to quantify reproductive tract maturation [13,44].

### 2.2. Experiment 1: Does Larval Diet Influence Adult Reproductive Status?

To examine how larval diet (in the form of host plant species) influences adult reproductive status and diapause, we assigned captive-raised, eastern North American monarch caterpillars to one of three host plant treatments: (i) greenhouse-grown native swamp milkweed (*A. incarnata)*; (ii) greenhouse-grown non-native tropical milkweed (*A. curassavica*); and (iii) field-grown swamp milkweed (*A. incarnata)* harvested in late summer. Tropical milkweed does not have a winter dormancy period, and is commonly purchased from popular garden centers and cultivated in gardens. Therefore, we assumed the tropical milkweed grown in the greenhouse shared some features of the tropical milkweed found in gardens (e.g., frequent watering and fertilizer application) that migratory monarchs encounter in the southern United States during the fall. Yet, we note that greenhouse plants will likely differ in leaf traits, chemistry, and growth rates from outdoor-grown plants. Greenhouse plants were grown from seed in a temperature-controlled greenhouse room (26 °C nighttime, 28 °C daytime), with artificial lights set to a 16-hr photoperiod to simulate growth conditions. Since host plant quality and senescence was previously shown to influence the induction of reproductive diapause in monarchs [13], the field-collected swamp milkweed from Athens, GA, United States was outdoor plants that had slowed in growth or declined in quality, collected from mid-August to September 2016.

The monarch caterpillars were the outcrossed, great-grand-progeny of wild monarchs from St. Marks, FL, United States (a migratory stopover site) collected during the fall migration in October 2015 and held to overwinter in the laboratory until late spring. We also included the grand-progeny of wild adults collected from Savannah, GA, United States, during summer 2016. Four genetic lineages of full- and half-siblings were used in the experiment. Eggs from mated females (screened to remove any infections by the protozoan *Ophyryocystis elektroscirrha*) were collected in a naturally lit room in August 2016 on the stalks of greenhouse-grown swamp or tropical milkweed.

On the day of expected hatch (3 days after egg laying), we transferred stalks to one of nine controlled environmental chambers (Model I-36VL, Percival Scientific, Perry, IA, USA), with three incubators for each of the respective host plant diet treatments (approximately 100 caterpillars for each diet group). The monarch caterpillars were reared under autumn-like conditions of cool temperature and decreasing day length, previously shown to induce adult reproductive diapause: 17 °C nighttime, 23 °C daytime, with a photoperiod of 13:11 light:dark, reduced by 2 min per 24 h [13,51]. Upon reaching the second instar stage, caterpillars were transferred from natal milkweed stalks to individual 0.5 L containers with mesh screen lids. Larvae were given fresh stalks (placed in florist tubes) of their respective diets every 1–2 days until pupation. For each individual, we recorded the number of days from hatch to pupation, mass (in mg) 2 days post-pupation, and number of days from pupation to adult emergence. 

Adults were transferred to a mesh cage (0.6 × 0.6 × 0.6 m) located within their natal environmental chambers, and continued to experience autumn-like conditions. We observed the adults twice per day to note mating pairs (pairs typically form late in the day and remain in copula for 12–16 h [52]). Because males initiate forced copulation in monarchs [53], we classified mated males, but not females, as “reproductive”, and unmated males as “not reproductive.” For males, we noted the number of days to first mating, removed them from cages, and placed them in glassine envelopes within the environmental chamber after the first mating event. The butterflies had *ad libitum* access to 20% honey water in petri dishes in their cages. Ten days post-emergence, we removed all adults from the environmental chambers and performed dissections (as described by Oberhauser and Hampton 1995 [54]). For males, we weighed the ejaculatory duct tract, previously shown to have lower mass when in reproductive diapause [13,22]. We categorized females with mature ovaries and fully chorionated oocytes (ridges present on oocytes) as “reproductive”, and those with small ovaries and lacking chorionated oocytes as “not reproductive” [44,55]. We also scored female egg development on an ordinal scale: (1) completely devoid of yolked oocytes; (2) presence of small yolked oocytes or non-chorionated oocytes; and (3) presence of at least one mature, fully chorionated oocyte.

### 2.3. Experiment 2: Does Adult Milkweed Exposure Affect the Reproductive Status of Wild-Caught Migratory Monarchs?

**Figure 1:**
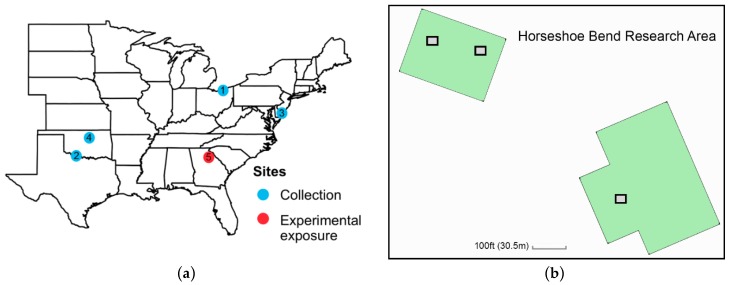
Map of eastern United States with collection sites and experimental exposure site along with schematic of the exposure site. (**a**) Migratory monarchs were captured at four stopover sites (blue symbols). Capture locations included (1) Cleveland, OH, USA (*n* = 123 monarchs) on 17 September 2015; (2) Burkburnett, TX, USA (*n* = 102) on 3 October 2015; (3) Cape May, NJ, USA (*n* = 168) on 12 October 2015; and (4) Stillwater, OK, USA (*n* = 106) on 13 October 2015. Experimental exposure to milkweed treatments in outdoor cages took place in Athens, GA (5; red symbol) at the (**b**) Horseshoe Bend Research Area of the University of Georgia. The research area includes pen grassy areas (green) along with three positions where cages were setup (gray boxes). Each cage held about 30 adults with 50:50 sex ratio.

To assess whether exposure to native and tropical milkweed alters the reproductive status of wild monarchs during fall migration, we captured wild monarchs at four stopover sites across the eastern United States (Figure 1a), where large quantities of adult monarchs were observed flying in a southerly direction, or displaying overnight roosting behavior, as reported by citizen scientists to the project Journey North [56,57]. Adult monarchs were captured using an aerial net between 09:00 and 17:00, and held individually in glassine envelopes at 14 °C for up to 72 h prior to being placed in outdoor flight cages. We captured approximately 100 adult butterflies per site from September to October 2015. All butterflies were transported to the University of Georgia (Athens, GA, USA) under permission from the United States Department of Agriculture (USDA; Plant Protection and Quarantine- 526 Permit # P526P-15-04201 to S. Altizer).

For each wild-caught adult, we recorded sex, body mass in g, and wing length in mm. We recorded lipid (fat) reserve score on scale of 0–4 based on the visual estimate of the thickness of an adult butterfly’s abdomen (0 represents very thin, with skin collapsing inwards, and 4 represents lipid-laden abdomen that appears swollen). We quantified wing damage on scale of 0–4 based on the number of wings with notable tears that might be caused by contact with a predator or hard surfaces. Wing wear was assessed on scale of 1–5, based on the loss of scales from the wings (1 represents newly emerged monarch with no scale loss, and 5 represents transparent wings with few scales remaining; following Cockrell, et al. [58]). The cumulative wing score was calculated based on the sum of wing damage and wing wear. Individuals with high wing scores (>5) were excluded, since these individuals were most likely older breeding adults originating from the summer season. We tested the adults for the protozoan parasite, *Ophryocystis elektroscirrha* (as described in Altizer et al. [59]), and excluded six infected monarchs from the experiment to avoid the confounding effects of infection.

From each collection site, we randomly assigned approximately 30 adults total, with similar number of males and females (50:50 sex ratio), to one of three treatment cages: (1) no milkweed (control), (2) native milkweed plants (*A. incarnata*), or (3) tropical milkweed plants (*A. curassavica*). The control in this study represented ambient fall conditions with no available milkweed for reproduction. The study was conducted in four replicate intervals, using adults from the four collection sites. Outdoor mesh flight cages (2 × 2 × 2 m) contained 12 potted milkweeds of either native milkweed or tropical milkweed (or no milkweed). All milkweed plants were grown in a greenhouse under the conditions noted for Experiment 1. Plants had approximately equal amounts of foliage and no flowers at the time of the experiment. Cages were set out in open grassy fields in the Horseshoe Bend Research Area of the University of Georgia (Athens, GA, USA) and arranged spatially to maximize the distance between the cages and provide a similar amount of sun exposure (Figure 1). For each replicate of the experiment (i.e., collection site), we randomly assigned treatments to three cage locations (Figure 1b), such that the spatial arrangement of the treatments with each replicate varied.

Butterflies were held in cages for 10 d and had *ad libitum* access to 20% honey water on sponges in petri dishes in their cages. We recorded daily high and low temperature and weather (as rain, sun, or overcast). We observed the adults twice per day (between 07:00–09:00 and between 17:00–19:00), which allowed us to detect mating pairs. We assessed reproductive status as described for Experiment 1. Males remained in the cages after their initial mating, to record the number of days to first mating and total number of matings over the 10 d period. We removed all individuals from the cages on the 11th day before noon, and dissected females within 48 h to record oocyte development. We note that reproductive status in this experiment was assessed in a more conservative manner for males (requiring successful mating) than females (based on oocyte development).

### 2.4. Statistical Analyses

#### 2.4.1. Experiment 1: Larval Diet and Reproductive Status

We used R programming software for all statistical analyses [60]. To examine whether larval diet predicted the reproductive status of adults, we first used a logistic regression model with a binary response variable (0 = no evidence of reproductive activity; 1 = reproductive). Predictors included host plant diet (with three levels), sex, and the interaction between diet and sex as fixed effects, as well as the environmental chamber replicate and monarch genetic lineage as random effects. Because the continuous covariates of monarch size and development rate (number of days from hatch to pupation, pupal mass, and number of days from pupation to adult emergence) did not differ between the diet treatments, these variables were not included in further tests (Table S1). Owing to potential differences in the costs of reproduction for females and males, and to examine measures of reproductive development (which differed for males versus females), we next analyzed data for the sexes separately. We asked whether the female egg development score was predicted by diet and mating status, with random effects of chamber and lineage, as before. We included mating status, a binary variable that indicated if the female experienced a mating event or not, as previous work indicates that mating can affect the maturity of oocysts [55]. We modeled the egg development score as a continuous variable and as an ordinal variable, and found qualitatively similar results (see Table S2); we report the continuous variable results in the main text. For males, we asked whether the mass of ejaculatory duct and the number of days to first mating were influenced by diet, again with chamber and lineage as random effects. Full model outputs of Experiment 1 analyses are available in Supplementary Materials (Tables S2–S4). 

#### 2.4.2. Experiment 2: Adult Migrant Milkweed Exposure

To examine whether the reproductive status of fall wild-caught monarchs captured as adults responded to milkweed exposure, we tested whether milkweed treatment (native milkweed, tropical milkweed, or no milkweed) and the interaction between sex and the treatment predicted reproductive status (as a binary variable), via a logistic regression model. Wing length, wing wear score, and fat score were included as continuous covariates. We included collection site and cage number as random effects, to account for any variation due to butterfly collection sites and positioning of the cages. Because preliminary analyses indicated that body mass was highly correlated with wing length and fat score (see Supplementary Materials for correlation matrix in Table S5), we did not include body mass in our final analyses [61].

Next, we analyzed reproductive measures for the sexes separately. We employed a mixed linear model to test whether the egg development score of wild females was predicted by milkweed treatment, mating status, and wing score, with collection site and position of the cage on the field as random effects. We modeled the egg development score as a continuous and ordinal (Poisson) variable and found similar results (Table S6); thus, we report only on continuous model results in the main text. Fat score and wing length were excluded, as they did not impact the egg development score of wild-caught females in preliminary analyses (Table S7). For males, we tested whether the number of mating events were predicted by milkweed treatment and wing wear score, wing length, and fat score, using a negative binomial model to account for zero-inflation (Figure S2), and included collection site and position of the cage on the field as random effects. We also used time-to-event analysis to determine whether treatment explained the timing to first mating event for males. We computed hazard ratios using a mixed-effects Cox proportional hazards model (R package coxme; [62]), where the event was first mating, and censoring was based on the observation of mating events. As before, random effects in the model included collection site and position of the cage on the field. Because collection sites were confounded with time, we did not include weather condition variables in our models, but report general trends in the Results below. Full model outputs of Experiment 2 analyses are provided in Supplementary Materials (Tables S6–S9).

## 3. Results

### 3.1. Experiment 1: Larval Diet and Reproductive Status

Of the 349 caterpillars at the start of the experiment, 295 (84.5%) survived to adulthood. Mortality of 15 caterpillars occurred in late instar stages due to unknown causes, while an additional 39 caterpillars died in the field-grown aged swamp milkweed treatment, owing to likely exposure to pesticides (nearby mosquito spraying) after showing signs of emesis and anorexia. Since the effect of pesticides on reproductive status in monarchs is unknown, we excluded 22 additional monarchs that were exposed to milkweeds from the same collection site from our analyses (resulting in a final sample size of 108, 98, and 57 monarchs fed on greenhouse-grown tropical (Tropical-GH), greenhouse-grown swamp (Swamp-GH), and field-collected swamp milkweed (Swamp-field), respectively). For summary statistics of days from hatch to pupation, mass post-pupation, and days from pupation to adult emergence per diet treatments see Table S10.

**Figure 2:**
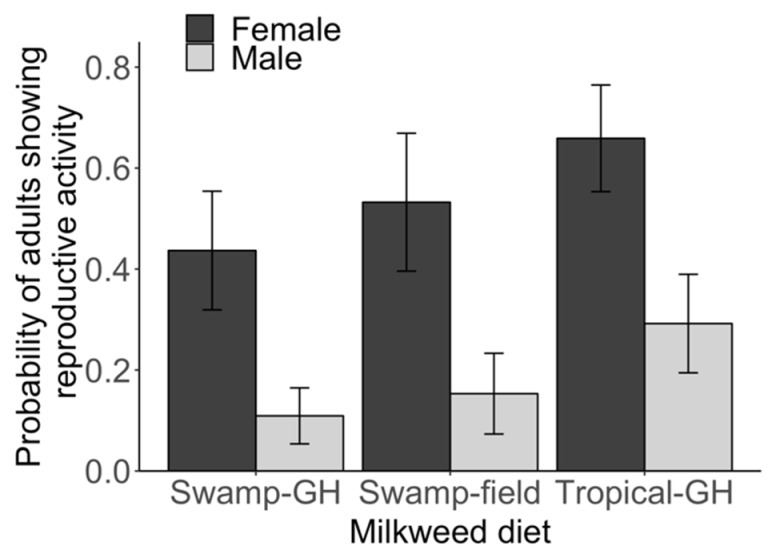
Reproductive activity in monarchs based on milkweed diet and sex. The probability that males and females show evidence for reproductive activity (mating in males, mature egg presence in females) as predicted by logistic regression model. Milkweed diets included “Swamp-GH”, which is greenhouse-grown native swamp milkweed (*A. incarnata*); “Swamp-field”, or field-collected swamp milkweed (*A. incarnata*) harvested in late summer; and “Tropical-GH,” greenhouse-grown, non-native tropical milkweed (*A. curassavica*). Monarchs fed tropical milkweed (*A. curassavica*) as caterpillars were more likely to show reproductive activity than those fed on native *A. incarnata* (either greenhouse-raised or field-collected). Females we more likely to show reproductive activity than males. Error bars represent ± SE. Full model output is reported in Table S3.

Among adults, 37% (97/263) were classified as reproductive based on male mating activity and female egg development. More females (55%; 70/128) than males (20%; 27/135) were classified as reproductive (Chi-squared = 14.87, *p* < 0.01; Figure 2). Our multivariate logistic model showed that the probability of being reproductive was lower in males (*z* = −3.41, *p* < 0.01) and higher with tropical milkweed diet (Tropical-GH: *z* = 2.10, *p* = 0.04); the interaction between diet and sex was not significant (*p* > 0.10; for full model outputs see Table S3).

Analysis restricted to females showed that egg development score as a continuous variable was not predicted by milkweed diets (Swamp-field: *t* = 0.04, Tropical-GH: *t* = 1.30, *p* > 0.05), or mating status (*t* = 1.09, *p* > 0.05; Table S2). For males, ejaculatory duct mass was not predicted by milkweed diet (Swamp-field: *t* = −1.73, Tropical-GH: *t* = 0.02, *p* > 0.05; Table S4a). Also, the number of days to first mating for males was not influenced by diet type (Swamp-field: *t* = −0.56, Tropical-GH: *t* = 0.25, *p* > 0.05; Table S4b). It is important to note that ejaculatory duct mass tended to be lower for monarchs that fed on field-collected swamp milkweed relative to other diets, but the difference was not significant (*t* = −1.73, *p* = 0.14; Figure S3; Table S4a). 

### 3.2. Experiment 2: Adult Migrant Milkweed Exposure

We captured 499 wild monarchs during the fall migration (Ohio: *n* = 123, New Jersey: *n* = 168, Oklahoma: *n* = 106, Texas: *n* = 102). Sex ratios were near even for the four sites, except for New Jersey, where we collected 63 females and 105 males. For this reason, we had a surplus of approximately 40 males from New Jersey that were not used for the experiment. Nine adults were excluded due to *O. elektroscirrha* infection. An additional eight adults were excluded owing to poor condition and high wing damage, suggesting that they were not migratory (*n* = 3 from Ohio; *n* = 2 from New Jersey; *n* = 1 from Texas), or because they died in transit (*n* = 2). Four adults perished during the experiment due to unknown causes. Weather conditions over the 10-day exposure periods experienced by the wild-caught monarchs differed somewhat for the replicates of the experiment (i.e., collection sites). Temperatures ranged between 3.9 °C (overnight low) and 32.2 °C (daytime high), with warmer temperatures during exposure for Ohio and Texas adults than New Jersey and Oklahoma adults, which were captured later in the fall (for dates and weather summary see Table S11).

**Figure 3:**
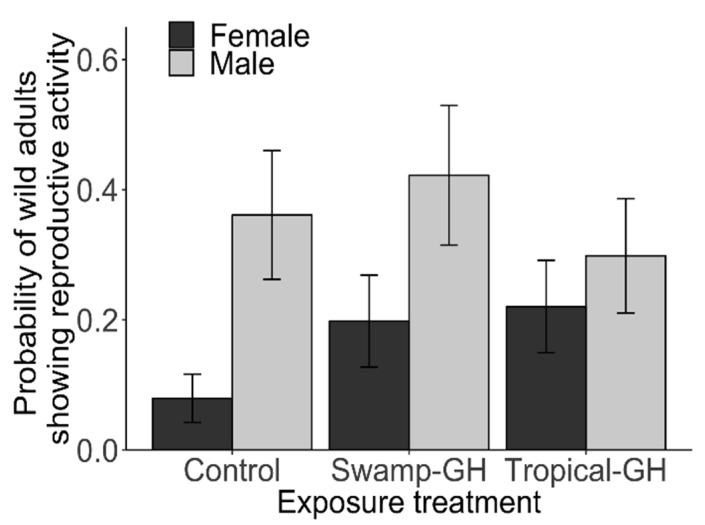
Reproductive activity status among wild-caught fall migrant monarchs under three exposure treatments. Exposure treatments in outdoor flight cages included “Control,” “Swamp-GH” (greenhouse-grown native swamp milkweed (*A. incarnata*)), and “Tropical-GH” (greenhouse-grown non-native tropical milkweed (*A. curassavica*)). Bars represent the model-predicted probability of reproductive activity. Females had a higher probability of showing reproductive activity following exposure to tropical milkweed plants compared to other treatments (for full model results see Table S8).

At the completion of the exposure experiment, 27% of adults (118 out of 438) were classified as “reproductive.” The proportion of adults that showed reproductive activity varied per collection site—34% from Ohio (*n* = 114); 26% from New Jersey (*n* = 123); 29% from Oklahoma (*n* = 104); and 17% from Texas (*n* = 96)—but differences between sites were not statistically significant (*F*_3,435_ = 2.80, *p* > 0.05; Figure S4). A higher proportion of wild-caught males (37%, 75/203) than females (18%, 42/235) were classified as reproductive (Chi-squared = 9.97, *p* < 0.01; Figure 3). Logistic regression showed that the probability of being reproductive (mating for males and presence of mature eggs for females) was higher for males (*z* = 4.09, *p* <0.001), and for those with higher wing score (*z* = 2.19, *p* = 0.03), such that individuals with greater scale loss and damage were more likely to be reproductive. The probability of being classified as reproductive for wild-caught adults was not influenced by fat score (*z* = 1.04, *p* > 0.05) or wing length (*z* = −1.44, *p* > 0.05). Finally, males in the tropical milkweed exposure treatment were less likely to be reproductive than males exposed to the other treatments (*z* = −2.44, *p* = 0.02; Figure 3, see Table S8 for full model output).

**Figure 4:**
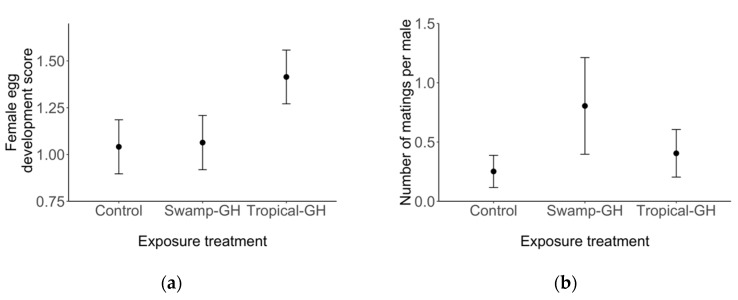
Reproductive traits of wild-caught monarch females and males in relation to milkweed exposure treatments. Points represent the predicted results of the linear mixed model. (**a**) Wild female migrants showed higher egg development scores following exposure to greenhouse-grown tropical milkweed (Tropical-GH), compared to native swamp milkweed (Swamp-GH), or to control treatments. (**b**) Males in the native swamp milkweed treatment (Swamp-GH) had higher numbers of matings than males in other treatments. Error bars represent ± SE. Full model outputs are reported in Tables S6 and S9.

Analysis restricted to females showed that egg development scores were higher in the tropical milkweed exposure treatment (*z =* 2.09, *p* = 0.04; Figure 4a) compared to other treatments, increased with wing score (*z =* 2.00, *p* = 0.05), and were higher for mated females (*z =* 3.00, *p* < 0.01; Table S6). Among males, analysis of number of matings per male showed an influence of exposure treatment, but wing length or score did not predict the number of matings per male (*p* > 0.05; see Table S9). Males exposed to native milkweed engaged in more mating events than males in other treatments (*z*= 2.54, *p* = 0.01; Figure 4b). Finally, the timing to first mating analysis (Cox proportional hazard model) showed that males in the native milkweed and tropical milkweed treatments had a higher probability of mating than males exposed to no milkweed (native milkweed *z* = 2.53, *p* = 0.01; tropical milkweed *z* = 2.14, *p* = 0.03).

## 4. Discussion

The results here show that monarchs exposed to tropical milkweed as larvae are more likely to be reproductively active, compared to monarchs exposed to native milkweed or no milkweed. When caterpillars were reared on tropical milkweed under autumn-like conditions, previously shown to induce reproductive diapause, both males and females were more likely to show evidence for reproductive development as adults, in comparison with monarchs reared on native milkweed. However, this effect was not apparent in sex-specific analyses of finer-scale reproductive development measures. Similarly, for fall migrant adults exposed to milkweed in flight cages, females showed evidence of reproductive development when exposed to tropical milkweed, relative to native or no milkweed. In contrast, male migrants exposed to native milkweed showed the greatest degree of reproductive activity, relative to tropical or no milkweed.

Previous work on diet and reproduction in other migratory insects showed that plant quality and secondary plant compounds can influence the maturation of reproductive organs in insect herbivores. For example, desert locusts (*Schistocerca gregaria*) fed old senescent leaves showed delayed sexual maturation, which was attributed to declining levels of plant hormones (auxins, gibberellins, and kinins) [63]. Work on tortricid moth larvae has shown that plant quality (protein concentrations) affects diapause induction [64], such that larvae that fed on higher quality plant species were less likely to undergo diapause induction at the larval stage. Work on swallowtail butterflies fed on high- versus low-quality host plants (of the same species) has similarly shown that low plant quality (tough leaves, older plants) was more likely to induce diapause in the pupal stage [41,65]. However, the degree to which host plant quality (including protein content and digestibility) versus the presence of specific chemicals (such as plant secondary compounds) are important for inducing or terminating diapause in insects remains unclear, and is likely species-specific [66].

In monarchs, milkweed plant age can also influence reproductive status, with larval diets of older milkweed leaves inducing higher rates of diapause compared to a diet of new milkweed growth [13]. We did not find this effect, possibly because the field-collected milkweed was chemically similar to the greenhouse-grown native milkweed diet. In particular, the native milkweed chosen here, *Asclepias incarnata*, has low concentrations of cardenolides and smooth leaves [40], and both young and older plants might be relatively nutritious and easy to digest [42]. How chemical composition changes as milkweeds age and enter dormancy is largely unknown, but could play a key role in induction of diapause in monarchs, potentially influencing juvenile hormone production. Future studies that include chemical analysis of the milkweed diets, particularly flavonoids, are needed to elucidate whether tropical milkweed lacks compounds that induce diapause, or contains components that influence levels of juvenile hormone and increase the probability of reproductive maturation. 

In agreement with previous studies, we found that a fraction of wild southbound monarchs during the fall migration period showed evidence of reproductive activity [30,46,67,68], possibly due to warm conditions monarchs can experience in the fall. For example, Satterfield et al. [30] found that 10 to 35% of wild fall migrants sampled in Texas showed evidence of reproductive activity, and Borland et al. [46] found that 14% of females sampled migrating through Texas had previously mated, as evidenced by spermatophore presence. Our experiment further showed that exposure to tropical milkweed was associated with a higher proportion of reproductive females that had fully developed reproductive organs and mature eggs present, compared to females exposed to native milkweed, or to no milkweed. Past studies of the effects of tropical milkweed on reproductive status of wild migratory monarchs found similar results: Batalden and Oberhauser [28] found that a small proportion of wild monarchs collected at an overnight roost during fall migration showed reproductive activity after being held in cages with tropical milkweed for 11 days. Satterfield et al. [30] found that wild migratory monarchs visiting tropical milkweed gardens in Texas had three times higher rates of reproductive development compared to migratory monarchs at stopover sites with no tropical milkweed. It appears likely that tropical milkweed advances the termination of reproductive diapause in migrating adults, resulting in the maturation of ovaries and production of eggs [41]. Tropical milkweed might have particularly high nutritional value or high concentrations of volatile compounds, such as flavonoids, that have been shown to stimulate female monarchs to produce mature eggs [42,69,70]. Although more detailed work is needed to determine the exact chemical stimulus responsible for sexual maturation in monarchs, our work adds to the growing body of evidence that planting tropical milkweed in eastern United States impacts southbound migration by influencing the number of monarchs with the propensity to breed.

Reproductive activity early in fall migration might be a bet-hedging strategy for migratory species. The timing and location of mating and egg laying are important in determining fitness, and if environmental conditions are favorable, engaging in reproduction rather than undertaking risky and energetically costly migration might be an option that yields higher fitness [1]. Partial migration, where both migratory and resident individuals are found in a population, is common in wildlife, with some fitness advantages associated with either strategy [71,72]. Warm fall temperatures, along with flowering and foliage-full tropical milkweed, likely signal to monarch butterflies the presence of a habitat that can support successful reproduction, ultimately contributing to the formation of resident subpopulations.

Wild-caught females in the adult exposure experiment showed a lower propensity for reproductive activity than wild males, regardless of the treatment, possibly owing to the costs of reproduction and migration between the sexes [73,74]. Specifically, females might experience higher energetic costs of egg development, locating suitable host plants, and oviposition [73]. Therefore, females likely require more resources and time to mature reproductively than males, even when cues to initiate reproduction are present. This idea is consistent with the observation of wild monarchs near the end of the overwintering period in Mexico, with males ending reproductive diapause several weeks before females [75]. Further evidence from monarchs and other insects suggests that males might be able to continue migration after attaining reproductive maturity [1,76]. Interestingly, the females in our larval diet experiment showed the opposite trend of the adult exposure experiment: regardless of the diet, females were more likely to be classified as reproductive compared to males. In addition, we found that the tropical milkweed diet produced more reproductive females than males. These results might provide an explanation as to why the sex ratio at the overwintering sites has become male-biased over than last 30 years [77]; if monarchs are increasingly raised on tropical milkweed as caterpillars, then fewer females emerge in reproductive diapause in the fall. Yet, it is important to note that we determined male reproductive activity in the diet experiment based on the observation of successful matings, and the actual number of males with mature reproductive organs may have been higher. Furthermore, the methods we used to assess reproductive activity were inherently different between males and females.

We found several interesting associations between physical characteristics of migratory monarchs and their reproductive activity. Specifically, females with more developed reproductive organs tended to have higher wing scores, which reflect wear of the wings as well as age. The simplest explanation for this pattern is that older females had more time to develop their reproductive system. Alternatively, older and more damaged females were less likely to complete the journey, and therefore initiation of reproduction was the better strategy. A similar explanation might apply to migratory males, which showed a significant negative relationship between the number of mating events and wing length. Previous work suggests that monarchs overwintering in Mexico have larger wings [67] and engage in mating events later than those with shorter wings [68].

Numerous questions remain regarding the impacts of tropical milkweed on the migratory monarch butterfly of eastern North America. While our work suggests that milkweed species can influence reproduction in wild migratory monarchs, we do not know the fate of the adults that end reproductive diapause and engage in reproductive activity. Previous work indicates that reproductively active monarchs, compared to those in diapause, are less efficient fliers and incur higher energy costs with flight [51]. Thus, we can speculate that successful migration to overwintering sites after breaking diapause is unlikely. A recent study examining the induction of reproductive diapause and the ability to orient towards Mexico in the fall showed that the two processes are not necessarily coupled [4]. Thus, what proportion of these monarchs attempt to continue their journey south to Mexico, or remain to reproduce at the sites with tropical milkweed, as well as how these activities might depend on other environmental conditions, are key questions that need further study (however, see Satterfield et al. [30]). Finally, tropical milkweed is naturally found in the lowlands of Mexico [32], yet sedentary monarch populations in these areas have not been reported, which suggests that additional factors, such as climate and the availability of foraging resources, might play a role in the successful formation of non-migratory monarch populations.

Further work is needed to explore how the underlying properties of different milkweed species, including plant chemistry and nutritional quality, influence monarch reproductive activity. Moreover, studies are needed to test how environmentally induced variation, such as plant age and herbivore activity, influence how monarch reproductive development responds to host plants. In particular, our experiments tested only greenhouse-grown tropical milkweed, rather than field-collected tropical milkweed grown under autumn conditions. Although tropical milkweed appears visually similar in autumn relative to plants during summer months (e.g., Figure S1b–d), changes in chemical properties, leaf traits, and growth rate in response to changes in photoperiod and temperature could occur.

Particularly interesting is the possibility of high fitness costs for monarchs that halt the migration to Mexico and instead remain in the southern United States to reproduce. Monarchs that remain in the United States face the risk of winter frosts and other unexpected weather events that can severely damage tropical milkweed plants, resulting in the starvation of caterpillars as well as mortality of the adults. Furthermore, recent work suggests that monarchs breeding during autumn and winter at tropical milkweed sites experience high risk of infection (prevalence reaching 100%) with a protozoan parasite, due to crowding [10,30]. This in turn poses an increased risk of infection to migratory monarchs in the spring as the migrants recolonize areas resided by the highly infected sedentary populations [30]. The sharp declines of monarch population size that have been documented at the overwintering sites in Mexico since the mid-90s [78,79] leads us to ask what the overall impact of planting of tropical milkweed might be on monarchs. The availability of tropical milkweed across the United States, particularly along their autumn migratory flyways (Figure S1a), might contribute to the loss of migratory monarchs (i.e., through migratory dropout) and lower the numbers of monarchs that ultimately reach the wintering sites in Mexico.

## 5. Conclusions

Planting of non-native tropical milkweed in gardens and parks along the migratory paths of the eastern North American monarch butterfly has been suggested to influence the formation of resident populations that breed year-round [10,80]. Here we show that tropical milkweed influences reproductive activity when larvae are fed this introduced plant species, and when female migrants are exposed in the field. Although successful migration and overwintering is dependent on multiple factors, the increased chances of becoming reproductive with tropical milkweed has important implications for monarch migration in the fall. These findings are not only pertinent for the southern United States, where resident year-round breeding populations form, but also for the northern United States, where the pool of monarchs that initiate and complete the migratory journey could be reduced. Conservation plans for monarch migration should therefore consider discouraging the planting of exotic tropical milkweeds in gardens and parks of the southern United States along the monarch’s migratory route.

## Supplementary Materials

The following are available online at https://www.mdpi.com/2075-4450/10/8/253/s1, Figure S1: Locations of tropical milkweed reports across the mainland United States; Figure S2: Histogram of wild-caught male mating counts; Figure S3: Male reproductive tract mass in relation to three milkweed diet treatments; Figure S4: Proportion of wild-caught (a) females and (b) males that showed reproductive activity based on collection sites following the experimental exposure; Table S1: Full generalized linear mixed model results for predictors of (a) pupal mass and (b–c) developmental times in relation to sex and milkweed diet treatment (Native-GH: greenhouse-grown native swamp milkweed; Swamp-field: field-grown swamp milkweed; and Tropical-GH: greenhouse-grown non-native tropical milkweed). Significant terms are presented in bold, *p* < 0.05. Random effects report the estimated variance and standard deviation for chamber ID and lineage intercept effects.; Table S2: Full generalized linear mixed model results for predictors of female egg development score as (a) continuous and (b) ordinal variable (with Poisson error) and (c) reproductive status in relations to milkweed diet treatment (Native-GH: greenhouse-grown native swamp milkweed; Swamp-field: field-grown swamp milkweed; and Tropical-GH: greenhouse-grown non-native tropical milkweed) and mating status (mated or not mated). Significant terms are presented in bold, *p* < 0.05. Random effects report the estimated variance and standard deviation for chamber ID and lineage intercept effects; Table S3: Full generalized linear mixed model results for predictors of reproductive status in relations to sex and milkweed diet treatment (Native-GH: greenhouse-grown native swamp milkweed; Swamp-field: field-grown swamp milkweed; and Tropical-GH: greenhouse-grown non-native tropical milkweed) as well as interaction between sex and diet. Significant terms are presented in bold, *p* < 0.05. Random effects report the estimated variance and standard deviation for chamber ID and lineage intercept effects; Table S4: Full generalized linear mixed model results for predictors of male (a) ejaculatory duct mass and (b) number of days to first mating event in relation to milkweed diet (Native-GH: greenhouse-grown native swamp milkweed; Swamp-field: field-grown swamp milkweed; and Tropical-GH: greenhouse-grown non-native tropical milkweed). Significant terms are presented in bold, *p* < 0.05. Random effects report the estimated variance and standard deviation for chamber ID and lineage intercept effects; Table S5: Pearson’s correlation matrix of fat score, wing score and wing length for wild-caught monarchs.; Table S6: Full model results for predictors of egg development score of wild-caught females as (a) continuous and (b) ordinal variable in relation to wing score, exposure treatment (Control: no milkweed; Native-GH: greenhouse-grown native swamp milkweed; Tropical-GH: greenhouse-grown non-native tropical milkweed) and mating status (mated or not mated). Significant terms are presented in bold, *p* < 0.05. Random effects report the estimated variance and standard deviation for collection site and cage position intercept effects; Table S7: Full logistic model results for predictors of all (a) adults’ reproductive status and (b) female egg development score (on continuous scale) in relation to wing length and fat score. Significant terms are presented in bold, *p* < 0.05. Random effects report the estimated variance and standard deviation for collection site and cage position intercept effects; Table S8: Full logistic model results for predictors of reproductive status of all wild-caught adults in relation to fat score, wing length, wing score, sex, exposure treatment (Control: no milkweed; Native-GH: greenhouse-grown native swamp milkweed; Tropical-GH: greenhouse-grown non-native tropical milkweed) and interaction between sex and exposure treatment. Significant terms are presented in bold, *p* < 0.05. Random effects report the estimated variance and standard deviation for collection site and cage position (as shown in Figure 1) intercept effects; Table S9: Full negative binomial model results for predictors of the number of mating events per male in relation to wing length, wing score and exposure treatment (Control: no milkweed; Native-GH: greenhouse-grown native swamp milkweed; Tropical-GH: greenhouse-grown non-native tropical milkweed). Significant terms are presented in bold, *p* < 0.05. Random effects report the estimated variance and standard deviation for collection site and cage position intercept effects; Table S10: Average monarch pupal mass, number of days from hatch to pupation, days from pupation to eclosion, female egg development score, male number of days to first mating, as well as proportion of individuals that showed reproductive activity in the three milkweed diet treatments: native greenhouse-grown swamp milkweed (Swamp-GH), native field-grown swamp milkweed (Swamp-field), and greenhouse-grown non-native tropical milkweed (Tropical-GH); Table S11: Collection sites of wild-caught monarchs along with collection dates, number of monarchs collected and approximate weather conditions experienced during exposure experiments. Daily temperature (°C) range reflects min and max over the 10 days of exposure; Table S12: Mean and standard error of wild-caught monarch female egg development score, number of days to first mating for males, number of matings per male as well as proportion of individuals that showed reproductive activity in the three exposure treatments: no milkweed (Control), native greenhouse grown swamp milkweed (Swamp-GH), and tropical greenhouse grown non-native milkweed (Tropical-GH).
