# Supplementary material for: Exposure to Non-Native Tropical Milkweed Promotes Reproductive Development in Migratory Monarch Butterflies
Source: Insects. 2019 Aug 16;10(8):253. doi: 10.3390/insects10080253 (PMC6724006; doi:10.3390/insects10080253)
Supplement: Supplementary file 1 [file insects-10-00253-s001.pdf]

Supplementary Materials

To accompany: Exposure to Non-Native Tropical Milkweed Promotes Reproductive Development in Migratory Monarch Butterflies (Majewska and Altizer 2019, Insects)

I. Sightings of Tropical Milkweed (*Asclepias curassavica*) across United States

We used the Global Biodiversity Information Facility (GBIF) [1] and iNaturalist [2] to acquire locations of tropical milkweed (*Asclepias curassavica*) in mainland United States from January 1999 to May 2019. We only searched for "human observations" and do not include preserved specimen reports from museum collections from GBIF; we only used "research grade" observation from iNaturalist. We found a total of 1635 occurrences of tropical milkweed with GPS coordinates.

References

1. *Asclepias curassavica* L. in GBIF Secretariat (2017). GBIF Backbone Taxonomy. Checklist dataset https://doi.org/10.15468/39omei Availabe online: https://www.gbif.org/ (accessed on 14 May 2019).
2. iNaturalist. Research-grade Observations. Occurrence Dataset. Availabe online: https://www.inaturalist.org/ (accessed on 20 June 2019).

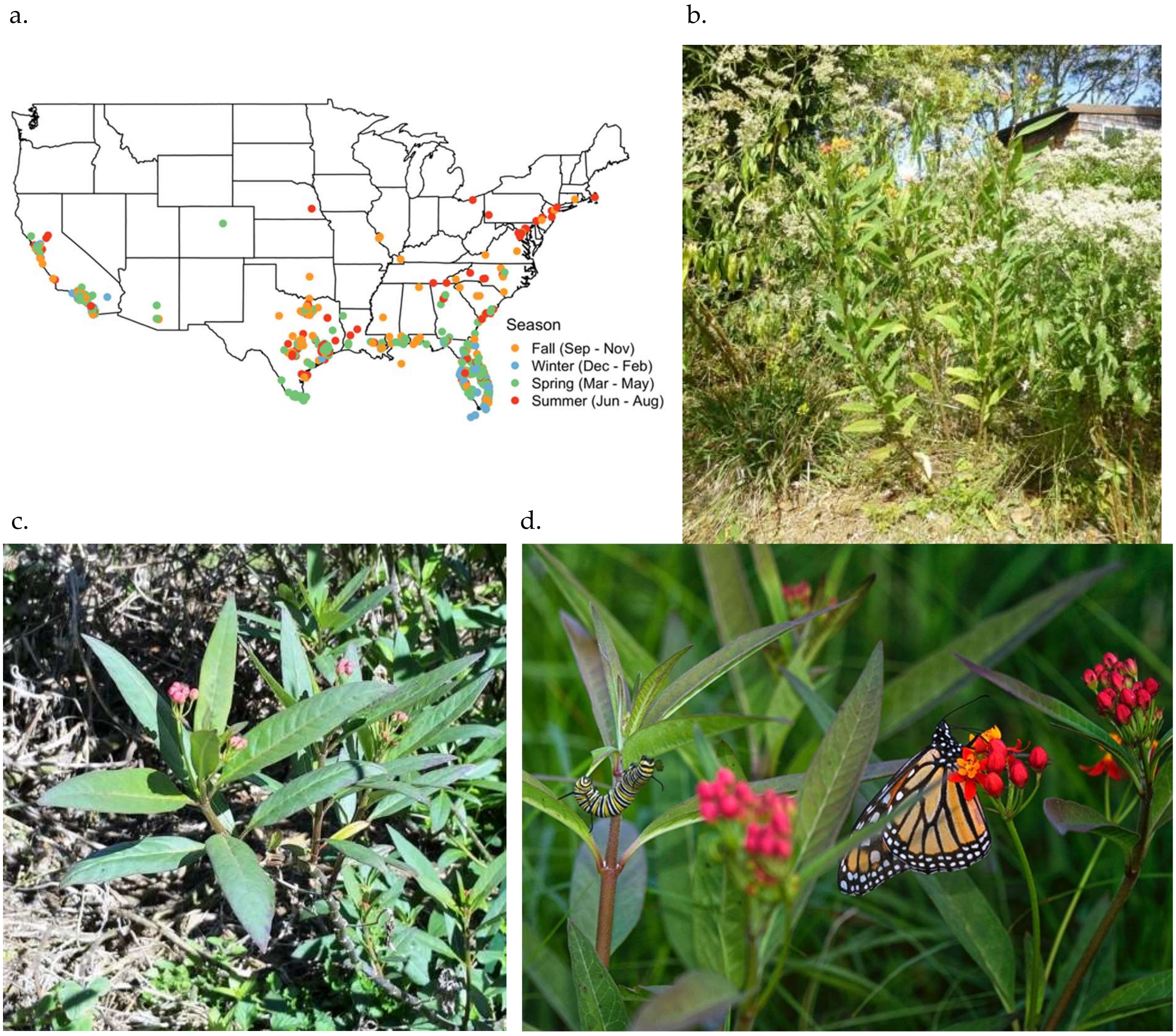

Figure S1. Locations of tropical milkweed reports across the mainland United States. (a) Locations of sites at which tropical milkweed (*Asclepias curassavica*) has been observed in the United States over the last 20 years. Filled symbol colors indicate four seasons, where orange represents autumn (Sep–Nov), blue represents winter (Dec–Feb), green represents spring (Mar–May), and red represents summer (Jun–Aug). While most sites are found in the southern latitudes of the country, a tropical milkweed plant was reported as far north as the state of Massachusetts. Tropical milkweeds are often found in gardens in autumn months, as exemplified by photographs from **(b)** Cape May, NJ in October 2015 (photograph by AA Majewska), **(c)** Johns Creek, GA in October 2018 (photograph by username *lja2* on iNaturalist), and **(d)** Savannah, GA in October 2014 (photograph by Fitz Clarke). Tropical milkweed persists and produces new leaves and flowers until a freezing temperatures damage or kill the plant.

II. Additional visualization

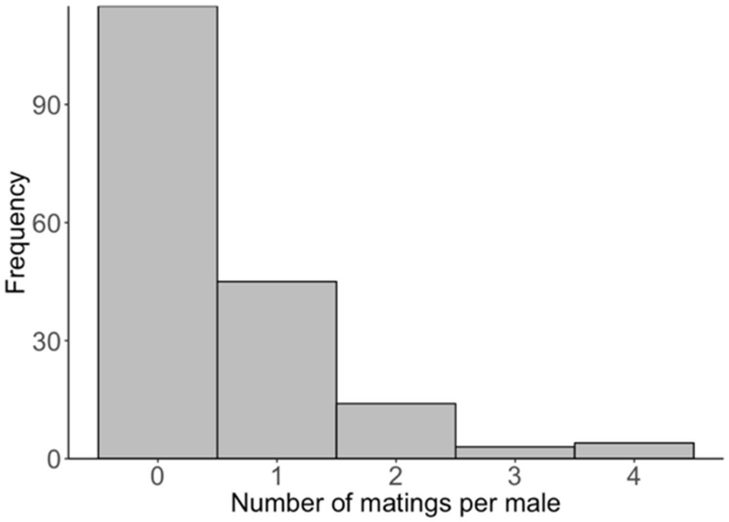

Figure S2. Histogram of wild-caught male mating counts. Distribution of the number of times wild-caught males mated.

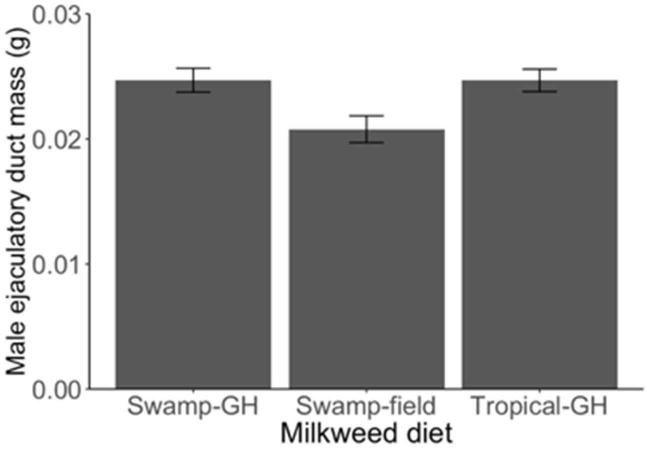

Figure S3. Male reproductive tract mass in relation to three milkweed diet treatments. Average monarch ejaculatory duct mass by three milkweed diets (Native-GH: greenhouse-grown native swamp milkweed; Swamp-field: field-grown swamp milkweed; and Tropical-GH: greenhouse-grown non-native tropical milkweed). Males tended to have lower mass of the ejaculatory duct when fed native milkweed compared to other diets, although the analyses which included random effects showed no significant difference between diet treatments (Table S4a). Error bars represent ± SE.

a. b.

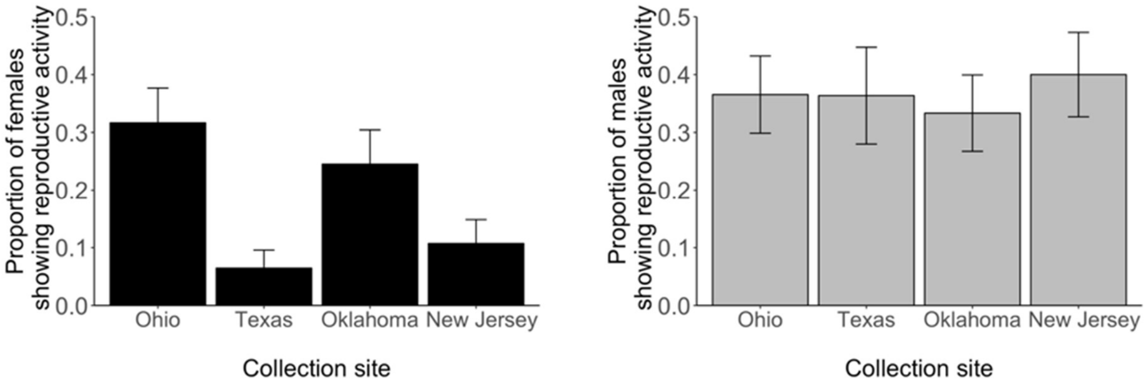

Figure S4. Proportion of wild-caught (**a**) females and (**b**) males that showed reproductive activity based on collection sites following the experimental exposure. Error bars represent ± SE.

III. Full Model Outputs for Experiment 1 (Larval Diet)

Table S1. Full generalized linear mixed model results for predictors of (a) pupal mass and (b–c) developmental times in relation to sex and milkweed diet treatment (Native-GH: greenhouse-grown native swamp milkweed; Swamp-field: field-grown swamp milkweed; and Tropical-GH: greenhouse-grown non-native tropical milkweed). Significant terms are presented in bold, $p < 0.05$. Random effects report the estimated variance and standard deviation for chamber ID and lineage intercept effects.

(a) **Pupal mass**	Estimate (SE)	t	*p*
Intercept	**1332.74 (81.80)**	**16.29**	**8.57e-07**
Sex (Males)	32.69 (68.55)	0.48	0.64
Swamp-field	−102.14 (119.10)	−0.86	0.43
Tropical-GH	−4.92 (105.58)	−0.05	0.97
Random effects	*Variance*	*Standard Deviation*	
chamber ID	8.033e+03	89.62518	
lineage	6.225e-05	0.00789	
**(b) Days from hatch to pupation**	Estimate (SE)	t	*p*
Intercept	**19.22 (0.52)**	**37.16**	**8.57e-08**
Sex (Males)	0.08 (0.18)	0.45	0.65
Swamp-field	1.18 (0.81)	1.46	0.20
Tropical-GH	−0.13 (0.72)	-0.18	0.87
Random effects	*Variance*	*Standard Deviation*	
chamber ID	0.706996	0.84083	
lineage	0.003052	0.05525	
**(c) Days from pupation to eclosion**	Estimate (SE)	t	*p*
Intercept	**15.44 (0.20)**	**77.42**	**5.40e-11**
Sex (Males)	0.43 (0.08)	5.51	9.34e-08
Swamp-field	0.19 (0.29)	0.65	0.54
Tropical-GH	−0.36 (0.26)	−1.40	0.22
Random effects	*Variance*	*Standard Deviation*	
chamber ID	0.08789	0.2965	
lineage	0.01888	0.1374	

Table S2. Full generalized linear mixed model results for predictors of female egg development score as (a) continuous and (b) ordinal variable (with Poisson error) and (c) reproductive status in relations to milkweed diet treatment (Native-GH: greenhouse-grown native swamp milkweed; Swamp-field: field-grown swamp milkweed; and Tropical-GH: greenhouse-grown non-native tropical milkweed) and mating status (mated or not mated). Significant terms are presented in bold, $p < 0.05$. Random effects report the estimated variance and standard deviation for chamber ID and lineage intercept effects.

**Females**			
**a) Egg development score (continuous)**	Estimate (SE)	t	*p*
Intercept	**1.20 (0.26)**	**4.70**	**0.01**
Swamp-field	0.01 (0.21)	0.04	0.97
Tropical-GH	0.23 (0.18)	1.30	0.25
Mated	0.17 (0.16)	1.09	0.28
Random effects	*Variance*	*Standard Deviation*	
chamber ID	0.01824	0.1351	
lineage	0.19687	0.4437	
**b) Egg development score (ordinal)**	Estimate (SE)	z	*p*
Intercept	0.15 (0.20)	0.75	0.46
Swamp-field	0.03 (0.22)	0.15	0.88
Tropical-GH	0.15 (0.18)	0.84	0.40
Mated	0.15 (0.19)	0.77	0.44
Random effects	*Variance*	*Standard Deviation*	
chamber ID	0	0	
lineage	0.08411	0.29	
**c) Reproductive status (0/1)**	Estimate (SE)	z	*p*
Intercept	-0.41 (0.55)	-0.74	0.46
Swamp-field	0.40 (0.53)	0.75	0.45
Tropical-GH	**0.95 (0.46)**	**2.06**	**0.04**
mated	0.26 (0.54)	0.49	0.62
Random effects	*Variance*	*Standard Deviation*	
chamber ID	3.584e-05	0.005986	
lineage	7.664e-01	0.875420	

Table S3. Full generalized linear mixed model results for predictors of reproductive status in relations to sex and milkweed diet treatment (Native-GH: greenhouse-grown native swamp milkweed; Swamp-field: field-grown swamp milkweed; and Tropical-GH: greenhouse-grown non-native tropical milkweed) as well as interaction between sex and diet. Significant terms are presented in bold, $p < 0.05$. Random effects report the estimated variance and standard deviation for chamber ID and lineage intercept effects.

**All adults**			
**Reproductive status (0/1)**	Estimate (SE)	z	*p*
Intercept	−0.25 (0.48)	−0.53	0.59
Sex (Males)	**−1.84 (0.54)**	**−3.41**	**6.58 e-4**
Swamp-field	0.38 (0.52)	0.74	0.46
Tropical-GH	**0.91 (0.43)**	**2.10**	**0.04**
Sex (Males) * Native field-collected	0.01 (0.84)	0.01	0.99
Sex (Males) * Tropical-GH	0.30 (0.69)	0.43	0.67
Random effects	*Variance*	*Standard Deviation*	
chamber ID	3.824e-10	1.956e-05	
lineage	5.235e-01	7.235e-01	

Table S4. Full generalized linear mixed model results for predictors of male (a) ejaculatory duct mass and (b) number of days to first mating event in relation to milkweed diet (Native-GH: greenhouse-grown native swamp milkweed; Swamp-field: field-grown swamp milkweed; and Tropical-GH: greenhouse-grown non-native tropical milkweed). Significant terms are presented in bold, $p < 0.05$. Random effects report the estimated variance and standard deviation for chamber ID and lineage intercept effects.

**Males**			
**a) Mass of ejaculatory duct**	Estimate (SE)	t	*p*
Intercept	**0.02 (0.01)**	**16.47**	**7.16 e-6**
Swamp-field	-0.01 (0.01)	-1.73	0.14
Tropical-GH	0.01 (0.01)	0.02	0.98
Random effects	*Variance*	*Standard Deviation*	
chamber ID	2.253e-06	0.001501	
lineage	2.346e-06	0.001532	
**b) Days to first mating**	Estimate (SE)	t	*p*
Intercept	**7.33 (0.57)**	**12.96**	**2.52 e-12**
Swamp-field	0.47 (0.84)	0.56	0.58
Tropical-GH	0.17 (0.66)	0.25	0.80
Random effects	*Variance*	*Standard Deviation*	
chamber ID	0	0	
lineage	0	0	
**c) Reproductive status (0/1)**	Estimate (SE)	z	*p*
Intercept	**-2.04 (0.43)**	**-4.69**	**2.7e-6**
Swamp-field	0.38 (0.65)	0.59	0.55
Tropical-GH	**1.23 (0.53)**	**2.32**	**0.02**
Random effects	*Variance*	*Standard Deviation*	
chamber ID	0	0	
lineage	3.47e-15	5.89e-08	

IV. Correlations between Morphometric Variables of Wild-Caught Monarchs

Table S5. Pearson's correlation matrix of fat score, wing score and wing length for wild-caught monarchs.

	**Wing score**	**Fat score**	**Wing length**
**Fat score**	-0.092		
**Wing length**	-0.062	0.236	
**Body mass**	-0.045	0.631	0.618

V. Full Model Outputs for Experiment 2 (Adult Migrant Milkweed Exposure)

Table S6. Full model results for predictors of egg development score of wild-caught females as (a) continuous and (b) ordinal variable in relation to wing score, exposure treatment (Control: no milkweed; Native-GH: greenhouse-grown native swamp milkweed; Tropical-GH: greenhouse-grown non-native tropical milkweed) and mating status (mated or not mated). Significant terms are presented in bold, $p < 0.05$. Random effects report the estimated variance and standard deviation for collection site and cage position intercept effects.

**Females**<br>**a) Egg development score (continuous)**	Estimate (SE)	t	*p*
Intercept	0.28 (0.33)	0.83	0.41
Wing score	**0.27 (0.14)**	**2.00**	**0.05**
Swamp-GH	0.02 (0.18)	0.13	0.90
Tropical-GH	**0.37 (0.18)**	**2.09**	**0.04**
Mated	**0.46 (0.15)**	**3.00**	**2.97 e-3**
Random effects	*Variance*	*Standard Deviation*	
Collection site	0.01904	0.138	
Cage position	0	0	
**b) Egg development score (ordinal)**	Estimate (SE)	z	*p*
Intercept	**-0.52 (0.25)**	**-2.13**	**0.03**
Wing score	0.18 (0.09)	1.94	5.27 e-1
Swamp-GH	0.02 (0.16)	0.13	0.90
Tropical-GH	**0.30 (0.15)**	**2.04**	**0.04**
Mated	**0.38 (0.12)**	**3.03**	**2.43 e-3**
Random effects	*Variance*	*Standard Deviation*	
Collection site	4.667e-03	0.0683176	
Cage position	1.245e-08	0.0001116	

Table S7. Full logistic model results for predictors of all (a) adults' reproductive status and (b) female egg development score (on continuous scale) in relation to wing length and fat score. Significant terms are presented in bold, $p < 0.05$. Random effects report the estimated variance and standard deviation for collection site and cage position intercept effects.

**All adults**<br>**a) Reproductive status (0/1)**	Estimate (SE)	z	*p*
Intercept	0.06 (2.63)	0.02	0.98
Wing length	−0.02 (0.05)	−0.46	0.64
Fat score	0.03 (0.15)	0.18	0.86
Random effects	*Variance*	*Standard Deviation*	
Collection site	0.08654	0.2942	
Cage position	0.14336	0.3786	
**Females**<br>**b) Egg development score**	Estimate (SE)	t	*p*
Intercept	1.85 (1.79)	1.04	0.30
Wing length	−0.01 (0.04)	−0.39	0.70
Fat score	0.01 (0.09)	0.14	0.88
Random effects	*Variance*	*Standard Deviation*	
Collection site	0.0186860	0.136697	
Cage position	0.0000221	0.004701	

Table S8. Full logistic model results for predictors of reproductive status of all wild-caught adults in relation to fat score, wing length, wing score, sex, exposure treatment (Control: no milkweed; Native-GH: greenhouse-grown native swamp milkweed; Tropical-GH: greenhouse-grown non-native tropical milkweed) and interaction between sex and exposure treatment. Significant terms are presented in bold, $p < 0.05$. Random effects report the estimated variance and standard deviation for collection site and cage position (as shown in Figure 1) intercept effects.

**Reproductive status (0/1)**	Estimate (SE)	z	*p*
Intercept	0.25 (2.84)	0.09	0.93
Fat score	0.17 (0.16)	1.04	0.30
Wing length	−0.08 (0.05)	−1.44	0.15
Wing score	**0.47 (0.22)**	**2.19**	**0.03**
Sex (Males)	**1.88 (0.46)**	**4.09**	**4.41e-5**
Swamp-GH	1.05 (0.56)	1.88	0.06
Tropical-GH	**1.19 (0.49)**	**2.40**	**0.02**
Sex (Males)* Swamp-GH	−0.79 (0.61)	−1.29	0.20
Sex (Males)* Tropical-GH	**−1.47 (0.60)**	**−2.44**	**0.02**
Random effects	*Variance*	*Standard Deviation*	
Collection site	0.04309	0.2076	
Cage position	0.24242	0.4924	

Table S9. Full negative binomial model results for predictors of the number of mating events per male in relation to wing length, wing score and exposure treatment (Control: no milkweed; Native-GH: greenhouse-grown native swamp milkweed; Tropical-GH: greenhouse-grown non-native tropical milkweed). Significant terms are presented in bold, $p < 0.05$. Random effects report the estimated variance and standard deviation for collection site and cage position intercept effects.

**Males**<br>**Number of matings per male**	Estimate (SE)	z	*p*
Intercept	2.59 (2.45)	1.06	0.29
Wing length	**−0.09 (0.04)**	**−1.98**	**0.05**
Wing score	0.28 (0.23)	1.23	0.22
Swamp-GH	**1.16 (0.45)**	**2.57**	**0.01**
Tropical-GH	0.47 (0.31)	1.52	0.13
Random effects	*Variance*	*Standard Deviation*	
Collection site	0.005567	0.07461	
Cage position	0.594206	0.77085	

VI. Additional General Results of Experiment 1 (Larval Diet)

Table S10. Average monarch pupal mass, number of days from hatch to pupation, days from pupation to eclosion, female egg development score, male number of days to first mating, as well as proportion of individuals that showed reproductive activity in the three milkweed diet treatments: native greenhouse-grown swamp milkweed (Swamp-GH), native field-grown swamp milkweed (Swamp-field), and greenhouse-grown non-native tropical milkweed (Tropical-GH).

	Milkweed diet treatment		
	Native greenhouse grown **(Swamp-GH)**	*Native field-grown* **(Swamp-field)**	*Tropical greenhouse grown* **(Tropical-GH)**
**Mean (±SE) pupal mass (mg)**	1350.38 (41.45)	1237.97 (39.62)	1347.44 (10.43)
**Mean (±SE) days from hatch to pupation**	19.36 (0.15)	20.51 (0.23)	19.33 (0.16)
**Mean (±SE) days from pupation to eclosion**	15.63 (0.06)	15.91 (0.12)	15.27 (0.06)
**Mean female egg development score**	1.24 (0.11)	1.39 (0.16)	1.45 (0.10)
**Mean male number days to first mating**	7.33 (0.23)	7.80 (0.30)	7.50 (0.17)
**Proportion reproductive adults**	26.5% (26/98)	35% (20/57)	47% (51/108)

VII. Additional General Results of Experiment 2 (Adult Migrant Milkweed Exposure)

Table S11. Collection sites of wild-caught monarchs along with collection dates, number of monarchs collected and approximate weather conditions experienced during exposure experiments. Daily temperature (°C) range reflects min and max over the 10 days of exposure. .

Collection site	Collection date	Adults collected (*n*)	Daily temperature range	Average daily temperature	Number of sunny days	Number of rainy days
Ohio	9/17/15	123	14.4–32.2	21.9	7	4
Texas	10/3/15	102	9.4–31.1	18.9	7	3
New Jersey	10/12/15	168	3.9–27.8	22.8	8	2
Oklahoma	10/13/15	106	3.9–27.8	22.8	8	2

Table S12. Mean and standard error of wild-caught monarch female egg development score, number of days to first mating for males, number of matings per male as well as proportion of individuals that showed reproductive activity in the three exposure treatments: no milkweed (Control), native greenhouse grown swamp milkweed (Swamp-GH), and tropical greenhouse grown non-native milkweed (Tropical-GH). .

	**Exposure treatment**		
	No milkweed **(Control)**	*Native greenhouse grown* **(Swamp-GH)**	*Tropical greenhouse grown* **(Tropical-GH)**
**Mean female egg development score**	1.06 (0.12)	1.05 (0.13)	1.40 (0.13)
**Mean male number days to first mating**	9.41 (0.19)	9.74 (0.16)	9.3 (0.22)
**Mean number of matings per male**	0.58 (0.09)	0.5 (0.11)	0.54 (0.11)
**Proportion reproductive adults**	25% (38/149)	25% (36/143)	29% (43/146)
